# Smoking cessation supported by a smartphone app: A qualitative process evaluation of the Quit Sense feasibility RCT

**DOI:** 10.1111/bjhp.70010

**Published:** 2025-07-25

**Authors:** Aimie Hope, Felix Naughton, Caitlin Notley

**Affiliations:** ^1^ School of Environmental Sciences University of East Anglia Norwich UK; ^2^ Addiction Research Group, Faculty of Medicine and Health Sciences University of East Anglia Norwich UK

**Keywords:** behavioural support, qualitative, smartphone, smoking cessation

## Abstract

**Objectives:**

Quit Sense is a Just‐in‐Time Adaptive Intervention (JITAI) smartphone app that provides real‐time automated and in‐situ support to help people attempting to quit smoking manage cue‐induced cravings. This process evaluation study explored views and experiences of feasibility trial participants and assessed: (1) intervention experiences, (2) how these might help explain causal pathways towards behaviour change and (3) experiences of study participation.

**Design:**

Qualitative interviews nested within a two‐arm feasibility randomized control trial.

**Methods:**

We purposefully sampled 20 participants (15 intervention, 5 usual care) for semi‐structured telephone interviews. Data were thematically analysed and was supplemented with a descriptive analysis of relevant experiences to hypothesize causal pathways to behaviour change.

**Results:**

Motivations for engaging in the trial and intervention included wanting greater accountability and to be part of something. Reasons for disengaging included successfully quitting (app no longer needed), lapsing/relapsing and preferring other support types. Mechanisms which reportedly enabled successful quit attempts included the app's prequit preparation phase through insights into smoking cues, the delivery of lapse avoidance strategies and the supportive messages which helped to reinforce the goal of quitting. The trial was conducted during the COVID‐19 pandemic and provided examples of situations and contexts in which Quit Sense was used and felt to be (un)helpful for cessation.

**Conclusions:**

The Quit Sense app and trial were well received by participants. Participants reported that the preparation phase used for app training prior to their quit date was of particular value and not currently offered by other apps tried.

**Statement of Contribution**

**What is already known on this subject?**

- Evidence indicates that the effectiveness of digital smoking cessation interventions is linked to how personalized and interactive they are.
- Digital cessation approaches that provide interactive on‐demand support to help address cravings are mostly reliant on user‐initiation of this support, and typically, smokers do not use this effectively.
- Just‐In‐Time Adaptive Interventions (JITAIs) that use sensors in smartphones to predict and respond to real‐time cravings without reliance on user initiation may help smokers manage difficult situations and contexts, but no studies have investigated user perspectives of this type of feature.

**What does this study add?**

- The Quit Sense app was seen as helpful by equipping users with strategies for resisting cravings.
- Quit Sense's prequit training phase was singled out as being of particular help for many.
- COVID‐19 movement restrictions meant some found the support less helpful over time and repetitive.

## INTRODUCTION

Smoking tobacco is the second largest contributor to the global disease burden and the largest single contributor to the UK disease burden (Allender et al., 2009; Murray et al., 2013). While quitting reduces the risk of many health problems, the success rate of those attempting to quit remains low, with over 75% relapsing within 12 months (Buss et al., 2023).

A major cause of smoking relapse is cravings brought about by environmental cues (e.g. seeing smoking‐related apparatus, other people who smoke or associations with a location), often referred to as ‘cue‐induced’ cravings. Cue‐induced cravings, learned primarily via operant and classical conditioning processes (Pavlov, 1927; Skinner, 1938), are implicated in almost half of all smoking lapses (any smoking) (Shiffman et al., 1996) and are not alleviated by the most commonly used cessation medications (Ferguson & Shiffman, 2009). Lapses to smoking, particularly in the first 2 weeks or so of a quit attempt, are highly predictive of long‐term relapse (Deiches et al., 2013; Kenford et al., 1994). For example, one large study found only 22% of smokers who lapsed early in their quit attempt (an average of 10 days after starting their attempt) were abstinent 6 months later compared with 71% who did not lapse early on (Deiches et al., 2013). Similar effects are reported when a lapse is experimentally induced early on in a quit attempt (Shadel et al., 2011).

Providing stop smoking support that is specifically designed to be effective in combatting cue‐induced cravings has been challenging (Naughton, 2017). Cue‐induced cravings are not alleviated by the most commonly used cessation medications (Ferguson & Shiffman, 2009). From a behavioural support delivery perspective, a challenge is that the time from craving onset to lapse is short (Ferguson & Shiffman, 2009) and, therefore, so is the window of opportunity to intervene. A further challenge is that people who smoke do not commonly proactively seek out support in the situations in which they are most vulnerable (Devries et al., 2012; Naughton et al., 2012). This means that interventions reliant on user‐initiated or on‐demand support (e.g. many apps, websites) are likely to miss the craving episodes which then lead to lapses (Naughton, 2017). App‐based interventions, however, are now seen to have the potential to offer solutions to these challenges, particularly Just‐In‐Time Adaptive Interventions (JITAIs) (Perski et al., 2022).

With Patient and Public Involvement (PPI) we developed, refined and piloted a theory‐guided smartphone app (Quit Sense, formally known as Q Sense) (Naughton et al., 2016) providing support to help people manage environmental cues to smoke as they arise. Quit Sense can provide ‘in the moment’ support that is relevant to the person and their location. Users train the app prior to making their quit attempt by reporting smoking incidences in context, including the presence of various smoking cues. Where reports are made more than once in the same location, a geofence (or virtual perimeter around the location) is created. Once the user's quit attempt starts, on entering that location and remaining there for between 1 and 15 min, or again after staying there for 3 h, support messages are triggered which are tailored to that location and the individual's location‐specific smoking cues. Pilot work has demonstrated that Quit Sense is both engaged with and acceptable to users (Naughton et al., 2016). Findings from a feasibility randomized controlled trial show high levels of uptake, positive engagement and support all of which indicate the potential of Quit Sense to support smoking cessation (Naughton et al., 2023).

The UK Medical Research Council recommends conducting qualitative process evaluations of trials to explain unexpected outcomes, contextual factors and to aid trial implementation (Moore et al., 2015). Qualitative data has an important role in capturing participant experiences of an intervention (e.g. ease of setting up and using the Quit Sense app) (Donovan et al., 2002). Qualitative methods can also provide insights into whether an intervention is functioning to change the individual's behaviour as intended (e.g. by revealing unanticipated or complex causal pathways) (Moore et al., 2015). This is pertinent to the present study where the Quit Sense app was tested by participants in real‐world situations. During this feasibility trial, participants interacted with Quit Sense in ways which were influenced by their personal circumstances, attitudes, beliefs, norms, resources and skills. As a result of these many variables, the intervention is likely to have differing outcomes for different users (Moore et al., 2015). Here, we report findings from a qualitative interview study nested within this trial. As part of interview analysis, we identified any descriptions given of either lapses or lapse avoidance in the context of the app. These instances provided insights into the contexts in which Quit Sense was operating and of the factors which may have strengthened or even impeded its intended effects.

The qualitative study had the following aims:

1. To gather user views on Quit Sense app usage to inform further optimization of the app.
2. To gain insights into potential causal pathways to behaviour change to refine the intervention logic model.
3. To understand participant experiences of the Quit Sense feasibility trial to inform future trial design.

## METHODS

A qualitative interview study nested within a two‐arm parallel group randomized controlled feasibility trial (Naughton et al., 2023), which allocated people who smoke at least 7 cigarettes per week, recruited online to either: ‘usual care’ (link to NHS SmokeFree website) (*n* = 105) or to intervention, who received ‘usual care’ plus access to the Quit Sense app (SMS link to app via Google Play app store with an initiation code) (*n* = 104).

Reporting adheres to the COREQ checklist for qualitative research (Tong et al., 2007).

The trial was conducted during the COVID‐19 pandemic, and participants' quit attempts were therefore made at a time when there were periods of national lockdown restrictions. In addition to capturing views of the acceptability of the app and study, the qualitative work captured insights into the effects of the pandemic on participants while they attempted to quit smoking.

### Participants

Participants were contacted after the trial's 6‐week follow‐up. We employed purposive sampling to achieve variation in socio‐economic status (individuals of high and low SES), app engagement (minimal to extensive) and smoking status (abstainers and those continuing to smoke). Interviews took place between 20 January 2021 and 22 April 2021.

### Quit Sense intervention

To provide context for this qualitative evaluation and the resulting interview findings, we provide a brief overview of the original intervention and evaluation in the Appendix S1. Please see the relevant publications for further details (Naughton et al., 2021, 2023).

### Data collection

Most eligible individuals were invited to interview by email (see Appendix S1), but some were invited by an SMS invitation or as part of a trial follow‐up phone call. Potential participants were provided with information about the interviews (e.g. duration, steps taken to preserve their anonymity) by the female Research Associate (AH), who had a doctorate in psychology and led on day‐to‐day study management, data collection and analysis. Information about the study (including interviews) was provided throughout, with a downloadable copy of the full participant information sheet being accessible via the study website. Consent to be invited to interview was given when participants enrolled in the study. While information on the interviewer's personal characteristics was not routinely provided (e.g. reasons and interests in doing the research), the researcher was able to answer any such participant questions at her own discretion.

The interview guide was developed in consultation with the public engagement panel. Interviews were conducted by the Research Associate (AH) over the telephone and were audio recorded. The mean duration was approximately 21 min. During the interview, intervention participants were asked about their experiences and views of self‐monitoring smoking using the app, including any environmental contextual factors/triggers missing from the smoking report survey which was part of the app. Participants were also asked about how easy the app was to use, the types of messages they liked most and least, the timing of messages when entering or dwelling in a smoking zone (geofence), views on features they would like that are not currently provided and how personalization of the app could be improved.

Both Intervention and Usual Care arm participants were asked about their experiences of participating in the study. Both arms were also invited to discuss their use or interest in the use of other smoking cessation aids and different types of cessation support available. Interview questions can be seen in the Appendix S1.

Interviews were transcribed to an ‘intelligent verbatim’ standard. All transcriptions were anonymized. All individuals who took part in interviews were compensated for their time with a £20 voucher.

Sample size was determined by incoming data during data collection and the extent to which additional interviews continued to provide insightful data (data saturation) and, ultimately, practical factors including the trial design and budget.

### Data analysis

Analysis and reporting of the interview data were focused on exploring participant engagement with the trial (all participants), use of the app (including any ways that positive behaviour change may have been facilitated) and instances of lapse or lapse avoidance (intervention group participants). A critical realist ontological stance was taken (Sayer, 2000). This recognizes that while there may be a singular ‘truth’ or reality, this can only be indirectly accessed through participants' own interpretations and representations of their experiences, which are shaped by the systems within which they are situated.

The analysis was led by AH who also designed and conducted the interviews. A thematic analysis approach was taken (Braun & Clarke, 2006). The researchers, therefore, looked to identify patterns of responses across the data set. The process followed is detailed below. The interviews were designed for the qualitative evaluation of the Quit Sense RCT. The ‘keyness’ of themes was therefore judged not only by prevalence across the data set but also by the extent to which they captured something important to participants' experiences of the trial, development of the app and experiences of smoking and cessation (Braun & Clarke, 2006).

First, AH began by listening through the audio recordings, followed by a phase of familiarization with the data, with AH reading each complete transcript. Then, AH began the process of generating initial codes. Coding was partially deductive to explore evaluation research questions, and conversations were shaped by the interview schedule. It was also inductive in that codes were developed from individual responses to the questions. Participants answered in their own words to describe their individual experiences of the trial, app, smoking and cessation attempt.

AH randomly selected 10 transcripts (7 intervention, 3 usual care). AH then coded each section of text to describe its contents, gathering all the relevant data within the selected transcripts relevant to each code for comparison. NVivo was used to facilitate the analysis. An initial coding structure describing themes and constituent codes was produced for testing on a further sample of transcripts. The aim of this was to see whether the patterns existed across the wider data set.

An iterative process of further developing and reviewing themes was then undertaken. A further 3 transcripts (2 intervention, 1 usual care) were randomly selected and independently coded by both AH and CN, with the two researchers meeting to compare coding. There was broad agreement. Based on the meeting, the initial coding structure was further refined and then used to guide the analysis of all transcripts, with changes being made to the coding structure as necessary. A further 2 transcripts were then independently coded by both AH and CN, with the researchers meeting a second time to compare coding. Thematic analysis was shared with the public engagement panel for member checking (Lincoln & Guba, 1985), thereby increasing the trustworthiness of the final analysis. Final adjustments were made to the coding structure, including defining and naming themes. The coding approach was then applied to all transcripts, so that all relevant extracts for each theme were collated and cross‐checked. A log containing field notes and reflections on findings was kept by AH, as recommended (Braun & Clarke, 2006). Together, these processes worked to help ensure that the coding was thorough and transparent in capturing the diverse content of the interviews.

As part of the main analysis AH recorded any instances within the interviews where participants talked either about lapsing or lapse avoidance in relation to use of the app. For example, instances where participants described using app strategies to manage strong cravings and identify and avoid smoking triggers were compiled, as were those instances where participants described the context in which they lapsed (e.g. drinking at Christmas). These excerpts were compiled to facilitate comparison and the content of each example, or ‘vignette’ was summarized along with its associated codes. The aim of this additional step in the analysis process was to help to identify potential pathways for behaviour change. Example exerts or ‘vignettes’ can be seen in Table S2.

Ethical approval for the study was granted by the Wales NHS Research Ethics Committee 7 (19/WA/0361).

## RESULTS

We invited 34 participants to interview and of these, 13 did not respond, 1 agreed but did not keep the appointment and 20 participated (5 usual care and 15 intervention arm).

The qualitative sample was broadly representative of the trial sample (Table 1) except that more qualitative participants had a degree, were of other‐than‐White ethnicity and were of a lower socio‐economic status. Both the latter were due to deliberative over‐sampling to ensure a broader range of views was captured in the qualitative study. One usual care and nine intervention participants were abstinent at interview. Results are presented below, organized by superordinate themes as headings.

**TABLE 1:** Qualitative sample in comparison with total trial sample.

	Interview sample (*n* = 20)	Trial sample (*N* = 209)
Age: mean (range)	39 (28–51) years	41 (18–61) years
Gender: *n* (%)		
Male	10 (50.0%)	93 (44.5%)
Female	10 (50.0%)	116 (55.5%)
Ethnicity: *n* (%)		
White	15 (75.0%)	191 (91.4%)
Indian	0 (.0%)	1 (.5%)
Pakistani	0 (.0%)	2 (1.0%)
Bangladeshi	2 (10.0%)	3 (1.4%)
Black African	0 (.0%)	2 (1.0%)
Black (other)	1 (5.0%)	1 (.5%)
Asian	0 (.0%)	3 (1.4%)
Mixed race	1 (5.0%)	3 (1.4%)
Not given	1 (5.0%)	3 (1.4%)
Socio‐economic status^a^: *n* (%)		
Low	10 (50.0%)	61 (29.2%)
High	10 (50.0%)	148 (70.8%)
Highest qualification: *n* (%)		
No formal	1 (5.0%)	13 (6.2%)
GCSE or similar	2 (10.0%)	42 (20.1%)
A/AS level or similar	2 (10.0%)	52 (24.9%)
Degree or similar	14 (70.0%)	90 (43.1%)
Other	1 (5.0%)	12 (5.7%)
Number of cigarettes usually smoke a day: mean (range)	14.7 (5–30)	15.4 (1–40)
Self‐reported as not smoking at 6 weeks (of those followed up): *n* (%)	10 (50.0%)	49 (33.1%)

^a^ Low socio‐economic status (SES) was defined as individuals who have a semi‐routine or routine and manual occupation (class 5 in the National Statistics Socio‐Economic Classification [NS‐SEC]) (Office for National Statistics, 2016), or who have never worked or are long‐term unemployed. High SES was defined as those in classes 1–4 of the NS‐SEC.

### ‘Connecting’: Reasons for engaging with Quit Sense

Participants were unconcerned about providing their personal details (e.g. telephone number, address) online to register for the trial. This was because they felt reassured by the references to a university and the NHS:

I've seen it's also in relation with the NHS […] I enrolled straight away […]. (Female, usual care arm [UC], ID110)

The main reported reason for participation was a desire to quit smoking, but related motivations included a desire for accountability, seeking extra support and wanting to make the quit attempt with others: ‘It was the accountability almost; just to be able to, you know, put a bit of pressure on myself rather that than just say, “I'm going to finish” and inevitably, I never do’. (Male, UC, ID116). Participants also commented that study‐related messages including telephone calls from the Research Associate helped them to feel involved and motivated (e.g. to complete follow‐up surveys).

### ‘Disconnecting’: Reasons for disengaging with the trial and app

This theme captures descriptions of where participants disconnected from the study and/or app, citing a variety of reasons including:

- Technical issues: A small number of participants reported issues with the app and associated frustration/annoyance. Issues included: unwanted and repeated battery optimization messages and crashing of other applications.
- Change in smoking behaviour: Some participants had uninstalled or disabled Quit Sense from their phones. Reasons included not making a quit attempt or lapsing from one, for example ‘when I started smoking again after that, I felt bad and embarrassed […] Just disengaged by that point’. (Female, Quit Sense arm [QS], ID210). Some reported disengaging because they had successfully quit cigarettes and/or transitioned to vaping.
- App a reminder of smoking: Interestingly, one participant who thought the app was very useful in the preparation phase ceased actively engaging once quit day arrived because they associated it with smoking: ‘One of the top tips [from the app] is to throw anything away that reminds you of your life when you were smoking. In my mind, that was also to get rid of the stop smoking app’. (Male, QS, ID216)
- Required other support: Some participants simply felt that digital support was not for them: ‘I removed it after a few days. I thought yeah, it's not going to work for me. […] To stop smoking it has to be at least a week or two of something physical […] Supervised [i.e., by a stop smoking counsellor] or patches’. (Female, UC, ID110)

### ‘Pathways for change’ quitting smoking with the Quit Sense app

Analysis of data collected from intervention group participants focused on app engagement, identifying ‘Pathways for change’ as a superordinate theme with subthemes reported below:

### The importance of ‘preparing’ to quit

‘Preparing’ was identified as a conceptually important theme. It related to Stage 1, which was the preparation phase of the app. This was valued as an opportunity to make a formal ‘commitment’ to quitting, including setting a fixed goal by establishing a quit date. This phase was seen as something novel, not being offered by similar apps, for example:

All the other smoking apps don't do that. On those ones, you've quit now. So, that was a nice one to be like, right, this is the day I'm going to quit, and it was that countdown […] it gave you that focus. (Female, QS, ID109)

Reporting appeared to assist some participants in reducing their cigarette intake during Stage 1. This reduction appeared to work via three main mechanisms. First, logging smoking helped to boost feelings of personal accountability and explore personal triggers. Second, the act of logging each smoking event helped to shift smoking from being habitual to being more of a conscious decision which could be questioned. Third, repeatedly logging smoking required effort, and, in some cases, it appeared easier to forgo the cigarette than to complete questions about the smoking event.

[E]very day that I smoke, I report it. With time I feel embarrassed, like I had someone watching over me. Yeah, so, I get to the point that I cut down during the time; instead of two cigarettes, I smoke one. (Female, QS, ID336)

[I]t firstly made me realise how many I smoked. […] when you're on the app and you see how many cigarettes you've had. Moreover, I would have a cigarette and then 10‐min later, I would have another one. Why? Why did I have that cigarette that I didn't need anyway and then half an hour later, I'll have another one? I found it useful, looking at that to see my own reasons for validating my own smoking. There was no hiding behind the ‘Oh, well, I've only had a few cigarettes’ […] it's in black and white. (Male, QS, ID425)

When I did need to go for a cigarette, I would do it without thinking about it. Using that app made me think about it a lot. Do I really need one? […] Every time I would have to go for one, I'd have to report it. So, in the end I was just thinking, I'm not going to go for one because I'd have to report it. That would then take my mind of wanting one. (Male, QS, ID206)

### The role of app messages in ‘validating’ quit attempts

App messages were largely deemed to be encouraging, supportive and motivational, offering validation of efforts to quit and helping to re‐enforce the goal of quitting:

I guess the main effect for me is in terms of it inspiring me […] every time I recorded that I'd had a cigarette, it would say something. Some of those ideas were new […] that encouragement was needed and definitely had an effect. (Male, QS, ID116)

Some participants described the app as a ‘positive voice’ and one which they felt able to internalize, for example:

Having the is like another positive sound in your head. It's something that's constantly encouraging you. (Female, QS, ID208)

While the information presented in the messages was not always new to participants, they reported finding the content provided a helpful reminder, for example ‘there's stuff that you know about but you kind of need to be reminded of it’. (Female, Quit Sense arm, ID106).

An example of how the context‐specific support messages delivered when within geofences were felt to be helpful includes a participant who tended to smoke while driving: ‘I would find, for example, on my way to work, the app would ping up saying, “you're in an area where you usually smoke.” It would then bring up a really useful tip on how not to smoke’ (Male, QS, ID216).

### Quit Sense helped to ‘equip’ participants with strategies

When asked, several participants recalled strategies suggested by the app that they had attempted, including breathing exercises, delaying smoking for a few minutes, using distraction techniques and removing visual cues to smoke:

I think the ones [app messages] that were just sort of saying, you know, “make sure you've cleared everything away beforehand.” So, I would make sure that the night before [Quit day], I would have my last smoke and then make sure that the tobacco I had then went away, out of sight. Got rid of the ashtrays. (Female, QS, ID114)

One participant explained how the app had equipped them with the knowledge of their smoking triggers, thus enabling them to quit:

When I started using the app, quitting for me, was always something that I would like to do but it just always felt like it's just that little bit out of reach. […] I very much attribute my quitting to the app because […][it] allowed me to unpick why I was smoking more. That was the key to the lock for me. (Male, QS, ID425)

### Impacts of timing and context on habitual smoking behaviours

Disruption to usual routines impacted both positively and negatively on quit attempts and included the lockdown during the covid pandemic and seasonal events such as Christmas, New Year and Ramadan.

#### Covid‐19

Individual differences were seen to shape responses during the pandemic, with participants responding in different ways to the lockdown. Some participants found ‘lockdown’ helpful in making a quit attempt because of, for example reduced social contact and increased financial pressures.

To be fair, it [lockdown] probably helped in a strange way, just because of the lack of socialising, which was kind of my key issue. (Male, UC, ID116)

Mostly, to be honest, my motivation to stop smoking was financial. I think I, like a lot of people, got made redundant towards the start of the pandemic. (Female, QS, ID106)

For other participants, however, the pandemic measures removed factors which had helped them to regulate their smoking. For example, spending more time at home rather than at work was an issue for one participant: ‘I knew as soon as I was working from home that I was getting bored […] When you're sat in a meeting for 2‐h; there's nothing much else you can do, so I'd think, “Oh, I'll just have a smoke” [laughs]’ (Female, QS, ID114).

#### Christmas and Ramadan

While study recruitment stopped between mid‐December 2020 and early January 2021 to avoid the main festivities of Christmas and New Year, a small number of participants commented that their quit attempts had been negatively impacted by timing:

Christmas and New Years' intruded within the time period that I'd set myself […] It came to Christmas and New Year and I found myself smoking and reaching for cigarettes even more rapidly than I had been. (Male, QS, ID120)

Some interviews coincided with preparations for Ramadan and Ramadan itself, with Muslim participants discussing how this was a positive factor—helping to motivate and enforce their quit attempt:

Ramadan is going to begin in less than 2 weeks. When you're fasting, you cannot eat, drink or smoke as well. So, I always look forward to Ramadan because it does help with stopping smoking. Because you're fasting, here in the UK, you'll be fasting for more like 10‐h a day. (Female, QS, ID208)

### Limitations due to the covid pandemic

Many participants trained/used the app during a period in which lockdown restrictions were in place across England. This restricted the number of locations participants accessed and which could therefore be recorded by the app and meant in some cases messages were repeated once all relevant ones had been delivered. For example one participant commented:

‘If you look at my records, it's mostly at home […] So, it didn't really teach me anything new about risky situations’ (Male, QS, ID299) and another reported that ‘Some of the things that come up, they do repeat quite a lot’. (Female, QS, ID315)

### Suggestions for app improvement

Participants made a range of suggestions for app improvement, including adding additional triggers as options when recording on the app, an option to make rapid smoking reports if time‐limited, inclusion of rewards to boost motivation when cigarettes are avoided and better tailoring by time of day (Table 2, including illustrative quotes). A summary of key findings that can inform future trial design and smoking cessation apps is provided in Table 3.

**TABLE 2:** Suggestions for app optimization with illustrative quotes.

Suggestion	Illustrative quote
Missing triggers (e.g. alcohol, daily routine, boredom)	‘Sometimes it can be as simple as after a meal’ (Female, QS, ID109)
Being able to report smoking quickly and while going between places (e.g. while walking or driving)	‘The ability to tell the app that you are driving, or for the app to recognize that’ (Male, QS, ID216)
Rewards to boost motivation	‘Every time you read a message maybe it could get something like a star’ (Female, QS, ID215)
Credit given for cigarettes not smoked	‘I always found it interesting to think back about “how many cigarettes have I not smoked?” So, if for example, the app knew that you were driving and then said afterwards that I would usually have 5 cigarettes on that journey, it could be that when you were driving it would say, “well done, that's 5 cigarettes you didn't have”’ (Male, QS, ID216)
Tailor messages by time, not just location	‘I liked the idea of the application training itself to my pattern and what I'm doing. But then I expected at the time that the application would identify the time that I was more likely to be longing for a cigarette and send me a message. So, I'm not sure if that is in the app or not but I would find that helpful’ (Female, QS, ID208)

**TABLE 3:** A summary of insights gained from the present trial which may be used to inform future trial design and smoking cessation apps.

Trial component	Considerations for trial and app design
Recruitment	Consider making any partnerships with academic institutions or health organizations explicit on advertisements to reassure participants of the trial's credentials. This reassured Quit Sense participants of the trial's validity
Retention	Consider having planned participant contact by a researcher or other team member. This helped participants to continue to feel involved and motivated The ability to provide technical support to resolve app issues
Trial timing	Be aware of the potential impacts of trial timing. Certain times of year may be more challenging (e.g. Christmas), while others such as preparing for and participating for fasting at Ramadan or the start of a new year may help facilitate behaviour change (see section on ‘disruption’)
App design	Consider having a preparation stage (prequit date). Participants found that making a formal commitment including setting a quit date, logging smoking events and noting personal contextual factors/triggers and preparation messages from the app was very helpful for their quit attempts Consider using geo‐location triggered tailored support to provide support for cue‐induced cravings

## DISCUSSION

Providing support to tackle cue‐induced cravings is a significant challenge given the brief time frame between craving onset and lapse. This qualitative evaluation enabled an exploration of experiences of using the Quit Sense app, a passively triggered JITAI which offers ‘real‐time’ proactive personally tailored and context‐specific support. Participants of the feasibility randomized controlled trial reported positive experiences of participation. Intervention participants reported that Quit Sense offered support in committing to a quit date, validating their intentions to quit via supportive messages and equipped them with a range of strategies in preparing for quitting, and resisting urges to smoke. Less positive feedback came from participants who had, for example experienced technical difficulties with the app or who felt that they needed a different type of support with their quit attempt.

The preparation (Stage 1) phase was singled out as of particular value and was felt to be a novel element of the app. Reporting the frequency, timing and locations of smoking reportedly helped participants to become more conscious of their habits and to understand their triggers. This aligns with prior qualitative work showing interest in tracking behaviour, including smoking, using apps to assist with behaviour change (Cerrada et al., 2017; Dennison et al., 2013). App‐based messages during the preparation phase were also seen as especially helpful in paving the way for a successful quit attempt.

One hypothesized pathway of behaviour change which was difficult to probe was the app's ability to provide context‐specific support using geofencing, which seeks to address Learning Theory targets. This function was impeded by lockdown when many participants' activity outside of their home was limited, impacting on work and socializing in particular. Nonetheless, the qualitative interviews provided insights into how the app may provide context‐specific support under more normal conditions. One interviewee, for example, had undertaken essential travel for work and had found that Quit Sense provided support in areas where smoking had previously been reported. Other participants, however, who were largely restricted to their home environments, felt app messages became less helpful over time and sometimes repetitive even though the app was expanded to include working from home as a ‘location’ and corresponding support messages. Finding support messages repetitive is reported with text message cessation systems, where the potential to adapt messages to changes in context are limited (Naughton et al., 2013), or where they are non‐tailored (Budenz et al., 2022).

The interviews also provided insights into some less expected pathways for behaviour change, although still broadly aligning with the theoretical underpinning of the app. For example, not all participants used Quit Sense as anticipated. This included disengaging with the app after the preparation phase because it was associated with smoking, and the user no longer identifying as a smoker.

The timing and context of individual quit attempts was observed to be an important theme within interviews. The disruption of the COVID‐19 pandemic and resulting restrictions on movement facilitated some quit attempts (e.g. financial pressures, reduced social contact), whereas other participants found themselves smoking more (e.g. while working from home). Adaptations in response to pandemic‐related disruption have been identified as key influencers of health behaviours (Notley et al., 2022). Negative disruption to habitual behaviours was also seen during the Christmas period, while positive disruption with the lead up to Ramadan was helped through engagement with the preparation phase of the app.

Qualitative process evaluation alongside feasibility trials is valuable for informing acceptability, feasibility and indicating necessary intervention changes prior to a definitive evaluation of the intervention, as per MRC guidance (Moore et al., 2015). This study supports prior findings on the feasibility and acceptability of Quit Sense (Naughton et al., 2016). Findings also align with those of a recent systematic review of digital smoking cessation interventions, finding that personalized or interactive digital cessation support (i.e. those tailored by participant responses or providing live feedback) was effective, whereas digital cessation interventions without such features were not (Fang et al., 2023). The review findings suggested that this was due to greater rates of engagement with the personalized/interactive interventions. Incorporating user views into app design and optimization is essential to further understand how best to promote engagement and therefore increase potential effectiveness for smoking cessation (Barroso‐Hurtado et al., 2021). More qualitative investigation into smoking cessation JITAIs is warranted.

Despite purposively sampling half of participants from the lowest (grade 5) Socio‐Economic Classification based on the NS‐SEC measure, most had a degree or higher as their highest educational qualification. Furthermore, the youngest participant was 28. This may mean we missed out on some valuable views and experiences among those with less education and a younger age.

## CONCLUSION

This qualitative study provides a range of insights into participant experiences of being in a trial testing a JITAI smoking cessation smartphone app. These included reasons for joining, such as seeking support and wanting to belong, and for disengaging, such as feelings of guilt at lapsing and no longer needing support. Participant experiences of using the Quit Sense app were generally positive, and they provided suggestions for further app optimization, such as further tailoring by time of day as well as location. The findings provide insights into potential causal pathways to behaviour change, including the importance of the app's preparation phase and reporting smoking episodes to inform intervention refinement.

## AUTHOR CONTRIBUTIONS

**Aimie Hope:** Conceptualization; methodology; data curation; investigation; writing – original draft; writing – review and editing. **Felix Naughton:** Conceptualization; methodology; supervision; funding acquisition; writing – review and editing. **Caitlin Notley:** Conceptualization; methodology; investigation; formal analysis; supervision; funding acquisition; writing – review and editing.

## Supporting information


Appendix S1

## Data Availability

The data underlying this article will be shared upon reasonable request to the corresponding author.
