# Supplementary material for: Smoking cessation supported by a smartphone app: A qualitative process evaluation of the Quit Sense feasibility RCT
Source: Br J Health Psychol. 2025 Jul 25;30(3):e70010. doi: 10.1111/bjhp.70010 (PMC12290499; doi:10.1111/bjhp.70010)
Supplement: Supplementary file 1 — Appendix S1 [file BJHP-30-0-s001.docx]

# Supplementary document for: Smoking Cessation supported by a smartphone app: A Qualitative Process evaluation of the Quit Sense feasibility RCT (Hope, Naughton and Notley)

Supplementary Table 1: Coding Framework

| Superordinate theme | Coding density (N=20) | Sub themes |
| --- | --- | --- |
| **“Connecting” – reasons for engaging with Quit Sense** | 17 transcripts, 45 references | Decision to take part:   - Finding out about the trial - Legitimacy (perceived) of the trial - Comments on the trial advertisement (for recruiting participants) - Comments on the trial website - Comments about signing-up (e.g., doing so immediately in case unable to find link again) - Wanting to try ‘something new’ (a motivation for joining) - ‘Accountability’ (a motivation for joining) - ‘Belonging’ (e.g., wanting to be part of a trial, a motivation for joining)   Overall positive comments on the trial |
| **‘Disconnecting’ – reasons for disengaging with the app and trial** | 18 transcripts, 139 references | - App was uninstalled, disabled, not activated, there was a decreased need for app support - Negative feedback on app messages e.g.,   - Did not like content or tone   - Message not appropriate for context or situation   - Messages became repetitive - Challenges in training the app by reporting smoking incidences e.g.,   - Did not have the opportunity to report smoking e.g., time pressures   - Phone was not accessible   - Travelling made it difficult to report - Technical issues (various) - Other and competing goals undermine engagement - Reporting was repetitive and a burden - App reminder of smoking |
| **Pathways for change** | 13 transcripts, 45 references | - Validating the role of app messages (‘Inspiring me’, ‘encouraging me’) - Tailoring – ‘it feels like messages are especially for me’ - App messages re-enforce goal of quitting - App encourages self-monitoring and self-awareness (especially via reporting smoking) - Self-regulation and self-efficacy boosted - ‘Stuck in my head’ (messages internalised) - Reduction in smoking due to being part of a study |
| **‘Preparing’ for quit day** | 10 transcripts, with a total of 16 references. | - Quit Date |
| **Time and context** | 19 transcripts, 44 references. | - Contextual factors outweigh support (e.g., home working) - Pandemic and current context - Time of year   - Christmas   - Ramadan |
| **Cessation as a process** | 11 transcripts, 27 references | - Assistance from Quit Sense made a difference (lapse and quit attempts) - Defensive response or justification given - Quit successful - Quitting concerns or side effects e.g., weight gain |
| **Smoking** | 19 transcripts, 54 references. | - Boredom - Identity - Discussion of lapses including previous quit attempts   - Cravings   - ‘Embarrassed’   - ‘Failure’   - Justifications   - Long term habit   - ‘One off’ / special occasion - Smoking as a reward or break - Smoking – general comments |
| **Suggestions from participants** | 13 transcripts, 53 references. | - App improvement suggestions e.g.,   - An ability to log or edit smoking reports after smoking incident   - Aesthetics   - App should count cigarettes not smoked   - Diary style function could be added e.g., ability to make notes on quitting journey   - Being able to specify what ‘other’ means   - A quick report function   - Add ability of app to recognise driving / travelling   - There are missing triggers / situations:     - Alcohol     - Boredom     - Habit   - Have rewards / badges |
| **Support accessed by participants** | 20 transcripts, 83 references. | - Strategies and aids including:   - Strategies suggested by Quit Sense (helped to ‘equip’ users)   - NHS Smokefree   - NRT   - Other apps   - Other strategies and aids   - Snacks, holding something   - Vaping / substitution |
| **App features** | 16 transcripts, 217 references. | - Comments on end of day app surveys e.g., Accountability, Convenience - Comments on different types of app messages e.g., daily, location triggered - Comments on app features such as ‘My profile and smoking patterns’ |
| **Control group (non-app participants)** | 5 transcripts (total of non-app participants), 20 references. | - Awareness of group allocation - Remaining motivated to participate - Thoughts about study materials - Study overall experience   - Devices used to access study   - Comments relating to RA and follow up contact   - Study communications |

Supplementary Table 2 Example Vignettes: Interview exerts illustrating lapse avoidance and lapsing in the context of the app

| **Successful quit** | | | | |
| --- | --- | --- | --- | --- |
| **ID** | **Summary** | **Examples of Codes applied to text as part of thematic analysis** | **Potential pathways / causes of change** | **Exerts from interview** |
| 267 | Used the app to help manage strong cravings and avoid lapses (e.g., by charting progress in My Profile and Smoking Patterns). Also employed strategies suggested by the app. | Time and context  Smoking  Cessation as a process  Preparation stage  Motivations  and justifications  Lapses including previous quit attempts | Increased financial constraints, health concerns.  Preparation phase and equipping – e.g., use of strategies suggested by app.  Self-efficacy boosted  Tracking progress using app, monitoring craving levels  Committing – joining a study, goal setting | I quit pretty quick, which I’ve not been able to do before. I’ve been smoking on and off since I was [in teens], and the last time I was able to quit was when I was pregnant.  *Interviewer: What do you think helped this time?*  Well, I think money, definitely. I lost my job […] that was definitely motivation to stop. […]  It’s an expensive habit. But also, my health in general was a huge motivation […] *Interviewer […] did you try out any of the strategies that the app suggested?*  I did the exercise ones, and I spent a lot more time indoors with my daughter. That was the strategy that said, ‘be indoors with people who don’t smoke.’ We don’t smoke inside, well, we don’t smoke at all now, but we don’t smoke inside the house, so I spent a lot more time like, forcing myself not to go outside. I also changed up my morning routine because we used to sit outside, have a cup of coffee, smoke and then go to work. So, my partner and I started exercising in the morning together and then we’d have a…well, I’d have a decaf coffee and then go to work, kind of thing […]  […] I was tracking my cravings and making sure that I was still on track. And noticing, okay, so I’ve only got three more days until you [the cravings] should start going away, sort of thing. You know, my cravings went from being really high to non-existent but it took about a week to actually get there. […] Just to remind myself that I did want to stop [laughs].  *Interviewer: So, from your survey, it seems that […] you haven’t smoked in the first two weeks of the study - how are things going now?*  I haven’t smoked at all. I have no desire to. I haven’t smoked since [….]. Well, I think the app gave me the kick up the bum. It made me make a commitment, ‘cause I’ve been umming and ahhhing about it for ages and just going, ‘Oh, I need to quit but you know, it’s the pandemic and I’m bored.’ [Laughs] I think it gave me…when you guys contacted me about using…well, I contacted you about using the app and then it said that I could use it, I think that gave me the push. It was something a bit more concrete and an actual set date and it really did keep me on track. |
| 347 | Participant actively engaged with the app. Attributed success to the app, at least in part. Training the app helped reduce smoking before making the quit attempt. Used strategies from the app to maintain the quit attempt and avoid lapse. App used most in Stage 1 (preparation) and then need for the app decreased. | Preparation  Cessation as a process  Support  Disconnecting /barriers to engagement  Reporting leads to reduction  Quit date (setting of)  Strategies suggested by the app | Preparing e.g., by reporting smoking. Boosted self-awareness of habit  Equipping e.g., use of strategies suggested by app | To be honest, I really thought I was going to fail at quitting but it [the app] actually works. I found that before the quit, when I had to log each of the times I was smoking, actually made me smoke less.  Yeah, I think it was that because if you smoked more than 5 in a day, you got a little sad face as well. So, I noticed as I was getting towards the quit date, I was actually thinking, do I need to really have this smoke or not? I was cutting down before which I think helps for when I did quit. I hadn’t been smoking as much just before then. […] Like I said, in the first week, I found it [the app] the most useful. I think I’d gone and brought a pack on mint imperials, so if I’m sat bored in a meeting, at least I can have one of those, to keep my hands and mouth busy for a few minutes. […] I was really, actually surprised that it worked [strategy suggested by app]. I’ve tried to do it before, and I failed within a week or two. So, it’s been really good. |
| 362 | Briefly used the app to identify where he smoked and then avoided those trigger locations. Attributed ability to quit both to lockdown and the app. | Drivers of engagement  Disconnecting/barriers to engagement  Time and context  Preparation  Smoking  Quit date (setting of)  Reporting smoking | Committing e.g., setting a quit date. Preparing for quit date. Learning about habits (used app to map and avoid contextual cues)  Social context supportive of quit attempt – a benefit of lockdown. | I like the…it didn’t last very long because I set a very short quitting date, but I like the idea of reporting where and when. I did that for a couple of days and then I purposely went to different routes; when I was on walk and that in the mornings, I would go on different routes to avoid that place. It became a routine that I would light a cigarette at that place, so I went a different way. […] So, I thought that was quite clever, recording where you’d had a cigarette and all that kind of stuff. […] I think lockdown has played a major part in it [cessation]. I’m not out and about in public or out socialising with my mates and all that kind of stuff. Because my wife doesn’t smoke and because I’m at home, it’s like, I just can’t be bothered with it. […]  I’m extremely happy about it. I think it’s brilliant. I’ve tried giving up at least, I’d say half a dozen times in my life and each time I’ve given up, unfortunately I’ve gone back for one reason or the other. Obviously, this time, I’m determined not to do. In previous attempts, I found it hard work. It’s not easy. All these people that say they found it a walk in the park…nonsense. In the past I’ve found it very, very difficult but this time, I’ve not struggled at all. |
| 425 | Used the app extensively in Stage 1 in multiple locations. Found the app increased awareness of smoking patterns and triggers and participant attributes this to being able to change their behaviour. Felt that the app messages had bolstered his motivation to maintain the quit. Gives an anecdote of when was about to lapse. This was prevented by message from the app which provides reminder that they are no longer a smoker. Largely disengaged with the app after doing the preparation stage because saw it as connected with old life as a smoker. | Cessation as a process  Support  Preparation  Cravings  Lapses / previous quit attempts  Messages – thoughts on  Strategies and aids  Self-efficacy  Reporting | Validating – app messages helped boost motivation to quit  Uses app to understand smoking patterns and triggers.  Reporting with app enabled this person understand their smoking patterns and triggers.  Change in self-identity – now a non-smoker | I think sometimes doing it for yourself or for someone else, isn’t always enough. Especially if you’ve got a really bad craving and this time around…I think I told you before that this time I haven’t been craving them whatsoever. But I know that in previous quit attempts, and there have been many. I would sit there with the cold sweats and the twitchy fingers going, ‘it’s okay, I’m doing something good for myself.’ I think you’re always after that extra bit of validation. I think a lot of the messages that are displayed through the app, they do just that. They’re not so much reminding you why you’re quitting. I think you know why you’re quitting and that’s why you’re quitting. What they do is they sort of they just bolster your defences a little bit. The odd fact here, the odd figure there. […] When I started using the app, quitting for me, was always something that I would like to do but it just always felt like it’s just that little bit out of reach. I just couldn’t quite grasp it. It was always something that I wanted to do but I couldn’t quite get there. Before the app, that was no different**.** I very much attribute my quitting to the app because it helped me subtract the patterns of me smoking. It allowed me to see when I was smoking more, and it then allowed me to unpick why I was smoking more. That was the key to the lock for me. It was helping me see that was why I was smoking more, when the only solution I could draw was because I felt I should. That really is a pathetic reason and it sort of broke down that barrier of not being able to quit. [….] I remember […] I was having a really bad day. […] I called into a services and I don’t know why because I’d never been there before in my life but I went and got a drink and I said, ‘I’ll take 20 [names cigarette brand] and a lighter,’ and that bloody app pops up with a little message saying, ‘have you thought about smoking today?’ And I immediately said, ‘do you know what, forget the cigarettes.’ The timing was absolutely impeccable. It was completely random because I’d never been in that services before. I have no idea why the message popped up, but it did. So as much as I say that I ignored the app after I quit – but perhaps I wasn’t thinking about smoking, I was just a bit stressed, I was a bit irritable. Then this message pops up. Even though it was completely nonsensical, it allowed me to take a deep breath and think, okay, so that message doesn’t make sense to my situation at the moment, but I was a smoker, I’m now not. I’m a lot better off for it. So, my app use definitely waned after I quit. In my mind, that was part of the design.  On the lead up to quitting, the way it tracks how you smoke, when you smoke, where you smoke. That doing that by itself is the reason that I do not smoke anymore. |
| 465 | Employed strategies suggested by the app to manage cravings and avoid lapsing. Engagement with the app was greatest in Stage 1 – preparing for quit | Preparation  Disconnecting  Strategies and aids / suggested by the app  Cravings  Vaping – substitution | Equipping user with app strategies  Preparation for quit stage particularly helpful  Substitution of smoking roll-ups with vaping  Disengaged with app as felt reduced need for support | I used the ones [strategies] that said, try and preoccupy yourself with something else. I found that if I happened to catch it at that point when I was having a craving then I did tend to find it helpful. *Interviewer: Have you tried any other quit smoking aids or support since you started the study?*  Yeah, I’ve been using my vape, so - but I haven’t been using any other apps or anything like that. […] Yeah, but I’m trying to sort of, ease up on it and ween myself down. Obviously, I’ve still got nicotine in the vape.  [….] I didn’t find it any less useful per say [in Stage 2], but I did find in that build up, it was more useful then in that build up. To be like, trying to ween down in this period and getting to the 1st of Feb when I set mine, then like I just found it…the further I got along with my quit attempt, the easier it was becoming. […] I found I had less reliance in the app itself, but I wouldn’t say I found it any less useful. |
| **Smoking / lapsed** | | | | |
| 420 | Engaged with the app by reporting smoking. This made the participant aware of their smoking patterns and triggers (e.g., boredom). They made the transition to vaping and have reduced the number of cigarettes smoked but have not completely quit. | Support  Smoking  Drivers of engagement  Boredom  Reduction  Vaping – substitution  Messages – thoughts on  Self-monitoring / self regulation boosted | Reporting promoted self-awareness of triggers and patterns  Substitution of cigarettes with vaping.  App equipped them with strategies (e.g., keeping hands busy)  Validating app messages, boosting motivation | It made me monitor pretty much everything in my life as well. Every time I needed to for a cigarette, like I say, I wouldn’t even think about it, I would just go. I was on 20+ cigarettes a day. At this moment in time, I’m on something called a vape. That’s mainly because it’s something to do with my hands. Now I have a cigarette in the morning, and I have a cigarette after my meal still too before bed. So, I’ve cut down from about 20+ a day to around 4-5 a day […] Normally it would be, oh, “I’m bored” or if I’m watching tv – “this is on an advert; I’ll just quickly nip out and have a smoke now” but it didn’t pop in my head. […] In the end, what I was doing was making a cup of tea or sandwiches or…just not thinking about going for a smoke. […] It’s about keeping my hands busy as well, it’s very difficult when you’ve been doing it for so long. I’ve been smoking since I was [a child]. I could probably stand with a smoker and not think about it. The smell might put us on the edge a little bit but like I say, I’ve always got my vape in my pocket. […]  All in all, the app is really great. Little messages really, really help. Especially when you know in the back of your head that you don’t need to go for one just because you’re bored. With the little messages there, for extra support, just telling you what your mind knows, that you don’t need to go for one. An app knows that you don’t need to go for one, so why are you going for one? |
| 218 | Did not feel ready to quit when quit date arrived and didn’t re-set quit date. Became demotivated and decided that the goal of quitting was not attainable within the time frame and disengaged. | Preparation  Time and context  Disconnecting / disengagement  Suggestions from participants  Quit date  Christmas / social situation outweighs support | Didn’t succeed in re-setting quit date  Stress, time of year/situation  Still smoking, discontinued efforts to quit | I set my quit date for as far in advance as I could because I wanted my quit date to be on like the 1st January, so it was like a New Year’s resolution. I don’t think it gave me the option to do that, so I had to put it on a few days before that, which was like just after Christmas. So, it would have been good to be able to do that. I think at the time of my quit date, it was when I was working over Christmas, so it was like a really stressful period. So, I was actually smoking more. So, I felt like a bit of a disappointed with myself and when I realised it wasn’t really possible in that time frame, so that’s when I kind of stopped using it. |
| 299 | Liked the app and reported smoking and set a quit date. In Stage 2 this person lapsed. This was over Christmas while socializing. Did report the lapses but largely disengaged with the app because considered that the attempt had failed. | Drivers to engagement  Disconnecting / barriers  Time and context  Support  Smoking  Christmas / social situation outweighs support  Reduction  Reporting encourages self-monitoring  Strategies and aids /inc. suggested by the app  Reporting smoking post quit date | Social situation outweighs cessation support  Saw lapse as a failure and discontinued efforts to quit  Attempted some app strategies  Disengaged with app support messages after reporting smoking post quit date | […] it was really useful and possibly even inspirational. Just the tips and the nature of it, you know, something there to aid you and encourage you to give up, it was a good thing. Also, that it had a real-world effect, it thought that it had a…yeah, it’s a good idea and it was a good experience. […]  [I] did start to tail off using it; the main reason for that is…the most risky situation for me to start smoking is either when I’m on my own and at a loose end, so kind of pottering around. Another one is, and even more so perhaps, socialising. Being with people. Christmas and New Years’ intruded within the time period that I’d set myself. I […] I found myself smoking and reaching for cigarettes even more rapidly than I had been, certainly more so than the early stages of me using the app. The app had a direct effect on reducing the number of cigarettes at first, probably for the first two or three weeks, so not really so long. Then towards Christmas, it would be too easy to pick up…and when I realised that I’d failed; I’d crossed the line, and I hadn’t given up. It just picked up again. […] Yeah, so I’d tried to go back to it [the app]. Like I say, sort of Christmas and New Year were what broke me away from it. I thought I’d go back to it for a few days or so and then it just began to tail off even more, so, yeah. It’s unfortunate that it was…for me, anyway, was around that time[…] But certainly, at the beginning, first two or three weeks, I was battling and every cig…it really does get me down because every cigarette that I have, I think about it and you want to just record the stress and thinking about what it’s doing to me and so on*.*  […] Yeah, I remember sort of delaying for a few minutes; I did do that, and it encouraged me to do that; smoking a cigarette is something that is often, not every time but often, I’d hesitate before doing it but then I would end up doing it anyway. So, there’s always that kind of delay.  *Interviewer: Yeah, I can see that. So, I can see that you’ve reported smoking post the quit date on the app. Do you have any memory of how helpful or otherwise you found those messages?*  By the time I got to that stage, I wasn’t really following the app, so I didn’t really take in those messages. I saw that it was giving messages, but I didn’t really read them to be honest. The main thing was that I was still smoking post the date. |
| 370 | Was smoking at the time of interview. Disengaged with the app after lapsing and deleted it. | Smoking  Disconnecting / disengagement  Embarrassed  Reporting smoking post quit date  Lapses including previous quit attempts  Messages – thoughts on | Negative emotions around lapsing  Saw lapse as a failure and discontinued efforts to quit  Disengaged with app support messages after reporting smoking post quit date | No, I used it for about a month. Of course, when I started smoking again after that, I felt bad and embarrassed, so I didn’t use it much after that but…  *Interviewer: Okay, did you still see messages and did you still report when you were smoking after the quit date?*  I did, yeah, and then after a couple of days, I deleted the app when I saw that it was using…’cause I didn’t see the point of using the data when I was back smoking.  *Interviewer: Okay and did you find any of the messages that it sent you when you’d started smoking again…did you find those helpful at all or had you disengaged by that point?*  Just disengaged by that point. Just more about, oh, another failed attempt. I’ll stop eventually [laughs].  *Interviewer: If you don’t mind me asking, what triggered you to restart smoking again?*  Funnily enough, just the worst time to stop smoking, shifts and things. ‘Cause I work for an agency, I don’t always get enough shifts and things, so that stresses me out. I’m thinking, ‘oh my god, how am I going to pay my bills?’ You end up smoking more and you shouldn’t be spending money on those things [laughs]. |

**Interview questions**

# Introduction / preamble to interviews

- Introduce myself
- Calling to get feedback on Quit Sense study. We really want to know about people’s experiences of this research so that this can be factored in when we design and run a much larger trial in future. Stress that there are no right or wrong answers and how important their feedback is
- Check aware that call will be recorded > that it will be written up, that personally identifying details (e.g., names, places) will be removed from the written version and the recording will then be deleted
- The call around half an hour but can be stopped at any point
- Reaffirm verbal consent

# Semi-structured interview topic guide

*Prompts are in blue.

## INTERVENTION ARM (Quit Sense - QS group)

1. Overall, how have you found using the Quit Sense app? *Has it met your expectations? Have you used the app as much as you thought you would? Why? Has that changed since you first installed it? [Probe for specific examples e.g. if not met expectations, can you say how? If a feature is liked, which feature, and can you give an example of how you used it or what you liked about it? Also probe for technical issues (installing, running, updates etc)?]

REPORTING SMOKING

1. How have you found reporting smoking using the app? * Did you use this as a tool for yourself? Were you honest? Did you feel like you were being checked up on? Is it easier to report in some places or at some times than at others? When reporting smoking are there any situations that are missing/hard to categorise?

MESSAGES

1. What are your thoughts on the messages the app sends you? *Do you feel the messages delivered during your quit attempt (if you’ve made one) have been appropriate for where you have been spending your time? Ask for an example. Are there any situations where messages have been particularly helpful/unhelpful? Ask for example. Why is that? Any differences PRE-QUIT + POST QUIT. * Did you rate the messages? If no, why not?

USE OF STRATERGIES AND AIDS

1. Which strategies suggested by the app (if any) did you use?  *Were there any app strategies which you found particularly helpful/unhelpful with managing cravings to smoke?
2. Have you used any quit smoking aids or other support since you started this study? In previous quit attempts? *If no – are you interested in using any? What have you used/tried? Examples - e cigarettes, other cessation apps, other health and wellbeing apps and the NHS website

END OF DAY SURVEYS

1. Did you complete the end of day surveys? What did you think of these? *E.g., in terms of timing and convenience. Any situations missing?

MY PROFILE [relevant IF they did the end of day surveys]

1. Did you view the My Profile feature? What did you think of it? *When did you look at it and why? Anything that could be added or improved?
2. Have you viewed the My Smoking Patterns feature? What did you think of it? *Did this tell you anything you didn’t already know about the things that make you want to smoke (e.g., places, moods, people, whether being aware made a difference to quit attempt). Example? *Have you noticed any changes to your triggers for wanting to smoke (i.e., cravings changed during the study?

LAPSES

1. Have you smoked at all (even a puff) during your quit attempt? If yes, what effect did it have on your quit attempt? *Did this affect your confidence in being able to quit, did you report this on the app and if so, was the app helpful? [>ASK ONLY IF FULLY RELAPSED > If you make another quit attempt in future would you use Quit Sense again?]

OVERALL APP (time allowing)

1. what one aspect did you most like about the app?
2. If you had to change one aspect of the app, what would it be and why?

STUDY EXPERIENCE

1. How have you found being part of this study so far? *Is it what you expected? Anything you think could be changed to improve your experience?

*It’s possible to provide study feedback using the audio-record function of the app. Have you seen/used this feature? How did you/would you feel about leaving recorded feedback?

CURRENT CONTEXT (time allowing)

1. Has your personal situation changed as a result of the coronavirus pandemic (probe, e.g. working from home)
2. Do you feel that the pandemic situation has impacted your smoking behaviour? Probe: more/less/different contexts of smoking? Aware of media reports around smoking and C19?
3. How have you found using the app during the pandemic situation? Probe: more helpful than it might have been, or less helpful than it might have been, messages tailored to situation or less relevant?

## USUAL CARE (SMOKE FREE- SF interviews)

INTRO along lines of > We are planning on doing a larger study in the future and we would therefore find your feedback on this study very helpful.

1. How have you found being part of this study so far? *Is it what you expected? Any suggestions on ways to improve the experience of participants?
2. Do you recall which group are you in (the app or the SmokeFree)? *How did you feel about being allocated to that group? Probe for awareness and understanding of randomisation. [Ask about ways to improve experience if people unhappy about allocation]
3. How did you decide whether to take part? *For example, did you talk to anyone about it, download the participant information sheet (PIS), did you enrol right away or return later?
4. What has helped you to remain motivated to stay involved in the study? *E.g., follow up calls, incentives, postcard. Have you felt as involved in the study as you would like to be? If not, is there anything we could do to make you feel more involved?
5. Am I right in thinking that you found about this study through seeing an online advertisement? What did you think about the ad? *Any concerns about following the link?
6. What did you think about the study website? *E.g., Was it easy to use and understand? Could you easily find all the information you wanted about taking part?
7. What did you think about the questionnaires? *Baseline and 6-week. Length, content, functionality on mobile/computer, time taken
8. What about the text messages you’ve received about the study? Have communications been too frequent, too few or about right?  *Or follow-up phone calls?
9. Since starting this study have you tried out any strategies to help you to stop smoking?
10. Have you used any quit smoking support? E.g., apps, medicines, self-help *Have you looked at / used the NHS SmokeFree website?
11. Do you have any other comments or suggestions? Anything you would like to ask us?

CURRENT CONTEXT (time allowing)

1. Has your personal situation changed as a result of the coronavirus pandemic (probe, e.g. working from home)
2. Do you feel that the pandemic situation has impacted your smoking behaviour? Probe: more/less/different contexts of smoking? Aware of media reports around smoking and C19?

## **Copy of interview invitation issued by email**

Dear [Name],

Thank you for volunteering to be part of the Quit Sense study. Without volunteers like yourself, this research would not be possible.

We’re inviting a small number of people to give feedback on their experiences of being part of the study and using the app. This feedback is important because it will be used when we design and run a much larger trial in future.

**Would you be interested in giving feedback?**Length is flexible but usually takes 15-20 minutes. To thank you we will give you a £20 Amazon gift voucher code.

What would giving feedback involve?

A telephone call which I will record. The recording will be typed up and anonymized so that any personally identifying information like names will be removed. We may use direct quotes when we share the findings, but these would not be linked to you.

Here are the types of things we would discuss [INTERVENTION GROUP]:

- Any feedback on the study (e.g., study communications, surveys, the extent to which you felt involved or not, what could be improved)
- How you have found using the Quit Sense app (what you liked most/least - don't worry if you didn't use it much - we'd like to know about that too)
- Any feedback on reporting smoking using the app
- Any thoughts on the messages the app sends
- Whether you used any quit smoking aids or other support since you started this study
- End of day surveys
- Features (e.g., My Profile, My Smoking Patterns, audio record)
- Feedback on experience of being part of this study so far
- Whether you’ve used any quit smoking support or strategies to help you to stop smoking
- Whether/how the pandemic has affected your smoking behaviour

OR

Here are the types of things that I will ask about [USUAL CARE GROUP]:

- How you’ve found being part of this study so far
- How you decided whether to take part
- How you found out about the study
- What has helped you to remain motivated to stay involved in the study
- Any feedback on the study website, questionnaires and text messages
- Whether you’ve used any quit smoking support or strategies to help you to stop smoking
- Whether/how the pandemic has affected your smoking behaviour

There are no right or wrong answers.  If there’s anything you don’t want to discuss, that’s totally fine, we can just move on to the next topic.

Please reply to this email to let me know whether you are interested / want to know more.

*If you are interested*, please feel free to suggest any days/times to talk that are especially good for you.

**Quit Sense Intervention**

To provide context for this qualitative evaluation and the resulting interview findings, we provide a brief overview here of the original intervention and evaluation.

A two-arm parallel-group randomised controlled feasibility trial was undertaken where participants smokers were allocated to ‘usual’ care (link to NHS Smoke Free website) or to receive ‘usual care’ plus the Quit Sense app. Follow ups took place 6-weeks after enrolment. This involved an online questionnaire, and the collection of the trial evaluation interviews which are presented here. A second follow up survey was collected after 6.5 months.

The main feature of Quit Sense the app is the ‘Geofence-Triggered Support’ (GTS) which is orientated around the following 3 stages:

Stage 1 is the training or preparation for smoking cessation stage (default 7-days). This includes making a formal resolution to quit marked by setting a quit date using the app. During this stage users report all smoking instances in their situational context and in real-time recording details such as their mood, location type, the presence of other people who smokedrs, etc. These details are used to tailor app support messages. If an app user reports smoking in the same location more than once a geofence is created representing a high risk area for that user. Geofences need to be larger (e.g., ~100m) than the location monitoring precision of the participant’s smartphone, which can vary between approximately 5 and 50m, depending on GPS/WiFi/network coverage. App messages at this stage focus on quit smoking cessation preparation, increasing motivation and self-efficacy.

Stage 2: the 28-day abstinence challenge. Post quit date the app monitors the user’s location and tailored support messages are triggered if/when a user enters a geofence area, based on the information provided in stage 1. App messages focus on quit re/lapse prevention.

In both stages 1 and 2 app users receive a non-tailored morning support message.

Stage 3: maintaining abstinence. The app sends messages for a further 2 months, but the frequency is reduced.

The design and content of the Quit Sense app (or intervention) are theory informed (using Learning Theory and Social Cognitive Theory).17-19 The app design is based on the premise that a smoker’s environment can trigger urges to smoke. It therefore targets learned associations between smoking and the physical environment, smoking and mood, and smoking and the presence of other people who smokers.

The app is designed to increase self-awareness of these kinds of smoking triggers. For example, self-monitoring of behaviour is promoted by using the ‘report smoking’ function with visual and graphical feedback. The app works to shape outcome expectancies (e.g., encouraging positive self-talk, imagining success) and provides a mechanism to formally establish a quit date (i.e., goal setting). It is designed to increase self-efficacy in resisting urges to smoke. This is done via provision of effective lapse-prevention strategies provided both by app messages and a ‘library’ of in-app advice (e.g., removing visual reminders like smoking apparatus before making a quit attempt, breathing techniques etc.).
